# Mitochondrial sequence diversity reveals the hybrid origin of invasive gibel carp (*Carassius gibelio*) populations in Hungary

**DOI:** 10.7717/peerj.12441

**Published:** 2021-12-07

**Authors:** Szilvia Keszte, Arpad Ferincz, Katalin Tóth-Ihász, Réka Enikő Balogh, Ádám Staszny, Árpád Hegyi, Péter Takács, Bela Urbanyi, Balázs Kovács

**Affiliations:** 1 Department of Freshwater Fish Ecology, Institute of Aquaculture and Environmental Safety, Hungarian University of Agricultural and Life Sciences, Gödöllő, Magyarország; 2 Department of Molecular Ecology, Institute of Aquaculture and Environmental Safety, Hungarian University of Agricultural and Life Science, Gödöllő, Magyarország; 3 Department of Aquaculture, Institute of Aquaculture and Environmental Safety, Hungarian University of Agricultural and Life Sciences, Gödöllő, Magyarország; 4 Fish and Conservation Ecology Research Group, Balaton Limnological Research Institute, Tihany, Magyarország

**Keywords:** Population genetic, Mitochondrial, *Carassius*, Hybridization, Taxonomy

## Abstract

**Background:**

Invasive gibel carp, *Carassius gibelio* (Bloch, 1782) has become well-established in the Hungarian waters and now are spreading in the European waters. On major concern now is the potential hybridization between gibel carp and the other invasive species in the *Carassius auratus* complex (CAC), which may further accelerate the spread of the whole invasive species complex. The identification of gibel carp and their hybrids is difficult because of its morphological similarity to the other species in CAC. Here we carry out a genomic assessment to understand the history of gibel carp invasion and its phylogenetic relationship with the other species in CAC. Three loci of the mitochondrial genome (D-loop, CoI, Cytb) were used to determine the phylogenetic origin of individuals and relarionship among six gibel carp populations and the other species in the CAC.

**Methodolgy:**

A total of 132 gibel carp samples from six locations in Southern Transdanubia (Hungary) were collected after phenotypic identification to measure the genetic diversity within and among gibel carp populations of Southern Transdanubia (Hungary). The genetic background was examined by the sequences of the mitochondrial genome: D-loop, Cytochrome *c* oxidase I (CoI) and Cytochrome *b* (Cytb). Mitochondrial genetic markers are excellent tools for phylogenetic studies because they are maternally inherited. Successfully identified haplotypes were aligned and with reference sequences in nucleotide databases (*i.e.,* NCBI-BLAST: National Centre for Biotechnology Information and BOLD: Barcode of Life Data System). The phylogenetic relationships among gibel carp populations were then analyzed together with the reference sequences to understand the relationship and the level of hybridization with the species in CAC.

**Results:**

Among the 132 aligned D-loop sequences 22 haplotypes were identified. Further examination of representative individuals of the 22 haplotypes, six Cytb and four CoI sequences were detected. The largest number of haplotypes of all three loci were found in Lake Balaton, the largest shallow lake in Central Europe. Based on the NCBI-BLAST alignment of the D-loop, haplotypes of *Carassius auratus auratus* and *Carassius a. buergeri* in CAC were identified in the *C. gibelio* samples. Further analysis of haplotypes with the other two mitochondrial markers confirmed the occurrence of intragenus hybridization of *C. gibelio* in the Hungarian waters.

**Conclusion:**

By using three mitochondrial markers (D-loop, Cytb, CoI), we genomically characterized a gibel carp-complex in Hungarian waters and assessed the *C. gibelio* phylogenetic status between them. Hybrid origin of locally invasive *Carassius* taxon was detected in Hungary. It points out that invasive species are not only present in Hungary but reproduce with each other in the waters, further accelerating their spread.

## Introduction

Gibel carp, *Carassius gibelio* (Bloch, 1782) is a highly invasive fish species in European freshwaters (Ferincz et al., 2016b; Piria et al., 2016; Puntila et al., 2013). It belongs to the Cyprinidae family, the biggest freshwater fish family, and the genus *Carassius* is native in East Asia (Nelson, Grande & Wilson, 1994). The circumstances of its initial introduction to Europe are still unclarified. The species’ mass invasion dates back to the second half of the 20th century (Balon et al., 1974; Banarescu, 1991; Holcik, 1980; Lelek, 1987), which was facilitated by human activities such as intentional introduction for creating aquaculture and accidental introduction to common carp stock (Kalous & Knytl, 2011) and their high ecological tolerance (Rylkova & Kalous, 2013) and ability of gynogenetic spawning (Zhou, Wang & Gui, 2000; Toth et al., 2000; Toth et al., 2005). Gibel carp was also introduced to North America (Elgin, Tunna & Jackson, 2014) and rapidly became one of the most successful invasive species, its area expanded by 233–1,250 km^2^/year (Docherty et al., 2017), which gives a warning sign to Western European countries, where gibel carp is occurring in an increasing number of freshwater habitats (Perdikaris et al., 2012; Vasile, Gibelio & Species, 2019; DeGiosa, Czerniejewski & Rybczyk, 2014; Przybyl et al., 2020; Verreycken et al., 2007). The species’ impact on native communities is mainly through food competition in natural waters (Halacka, Luskova & Lusk, 2003). For example, it is known to outcompete crucian carp (*Carassius carassius,* Linnaeus 1758), native to European lentic waters (Harper et al., 2019).

In Hungary, it was mentioned in the literature the first time in 1887 by Herman, but it was most likely a misidentified specimen (Herman, 1887). The first official shipment to fish farms arrived in 1954, from Bulgaria (Szalay, 1954). Nowadays it is one of the most common generalist fish species in the lowland waters of the country (Takacs et al., 2017).

Although gibel carps are now found in most of European waters, its taxonomy remains unsolved. The species has been grouped into the *Carassius auratus*-complex (CAC), which also includes, for example, *C. auratus*, *C. gibelio*, *C. praecipuus*, *C. langsdorfii*, *C. cuvieri*, *C. carassius* (Rylkova et al., 2018; Takada et al., 2010). Some authors referred it as a subspecies of the goldfish, *Carassius auratus* (Linnaeus, 1758), but the others argued that the differences are not enough to list it as a subspecies and both belong to the same species *C. auratus* (Berg, 1932; Guti, 1993; Pinter, 2002). Furthermore, gibel carp has different levels of ploidy (2n = 100, 3n = 150–160) within a single population (Zhou & Gui, 2017) which makes the genetic diversity analysis of this species even more difficult. Kalous et al. (2012) have specified two neotypes of the *C. gibelio*. The described neotypes and knowledge of the genetic background allows tracking of the spreads of gibel carp populations and CAC and help understand the level of hybridization among them. Hybrids between gibel carp and the other species in CAC can be more of a threat to native species than gibel carp in European waters given their rapid growth and even wider environmental tolerance (Wouters et al., 2012).

The main objectives of this study are, by using different molecular markers, to identify gibel carp haplotypes, assess the phylogenetic status of the recent, locally invasive populations in Hungary, and understand their genetic diversity within and among these populations.

## Materials and Methods

### Sample collection and DNA extraction

**Figure 1:**
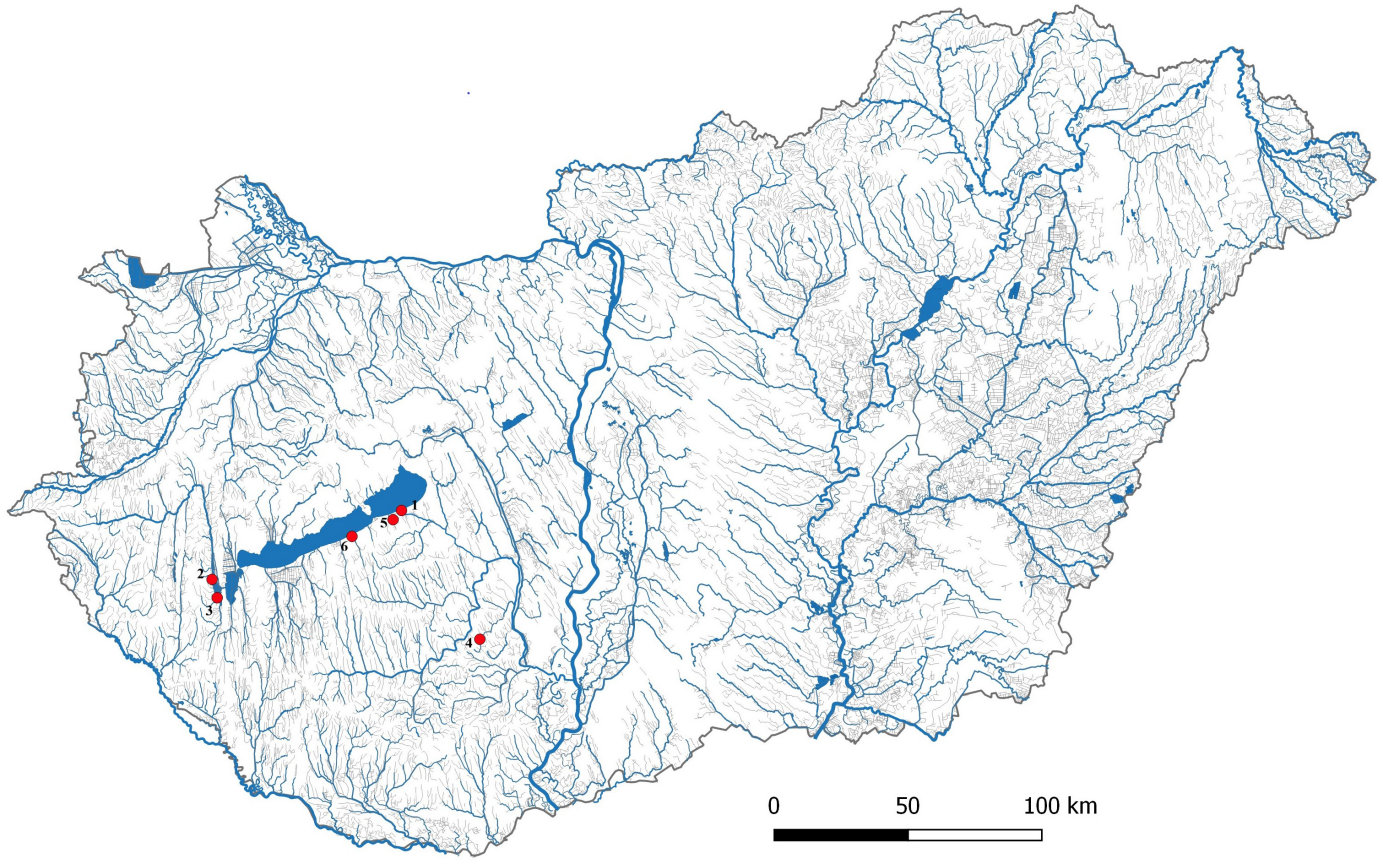
Location of Carassius gibelio sampling sites. 1:Siófok, 2:KWBPS II., 3:KWBPS I., 4:Hőgyész, 5:Siófok-Törek, 6: Őszödi-berek.

One hundred and thirty-two gibel carp samples were collected from six locations in Hungary including Lake Balaton (Siófok; *n* = 29; N46°54′24 E18°02′41), two reservoirs of Kis-Balaton Water Protection System (KBWPS) I stage (*n* = 17; N46°36′02 E17°09′01) and II (*n* = 18; N46°39′47 E17°07′23), Hőgyész (*n* = 30; N46°28′34 E18°26′02), Siófok-Töreki fish pond system (*n* = 19; N46°52′32 E18°00′14) and Őszödi-berek wetland (*n* = 19; N46°49′02 E17°48′12) (Fig. 1). Fish collection for laboratory examinations was authorized by the Government Office of Pest county (Permit no.: XIV-I-001/2302-4/2012). Fishes were collected with electrofishing and from the eel trap of Balaton Fish Management Nonprofit Ltd. at Siófok. Since gibel carp is an invasive fish species, all collected individuals were euthanized with clove oil before tissue samples were collected for DNA sequencing. Tissue samplings have been authorized by the Minister of Agriculture (Permit no.: HHgF/122-1/2018). Tissue samples were collected from the caudal fin and then stored in 96% ethanol at −20 C° before use. DNA was isolated from the tissue samples by E.Z.N.A Tissue DNA Kit (Omega Bio-tek, Norcross, GA, USA) following the producer’s protocol. DNA concentration was measured by spectrophotometer (IMPLEN, NanoPhotometer™ Uv/Vis) and the quality was tested by running 250 ng of each DNA sample on 1.5% agarose gel.

### PCR amplification and sequences

The control region of the mitochondrial genome (D-loop) was amplified with primers from the Cyprinidae family, Carp-pro2-F (5′-TCACCCCTGGCTCCCAAAGC-3′) and Carp-phe2-R (5′-CTAGGACTCATCTTAGCATCTTCAGTG-3′) (Wang et al., 2010). PCR reaction final volume was 25 µl, contained 1  × PCR buffer with (NH_4_)_2_SO_4_ (Fermentas; Thermo Fisher Scientific, Waltham, MA, USA), 2000 µM dNTP mix, 250 nM for each primer, 1.5 mM MgCl_2_, 100 ng template and 1 U Taq polymerase (Fermentas). To collect more information about the populations, the resulting haplotypes were analysed by using the same protocol with primers from the Cytochrome *c* oxidase I gene (CoI): CO1_FF2d_F (5′-TTCTCCACCAACCACAARGAYATYGG-3′), CO1_FR1d_R (5′-CACCTCAGGGTGTCCGAARAAYCARAA-3′) (Ivanova et al., 2007) and with primers from the Cytochrome *b* (Cytb): Cytb_H_2_R (5′-GTTTGTTTTCTAACCCGATCAATG-3′) Cytbas_F (5′-GAAGGCGGTCATCATAACTAG-3′) (Xiao, Zhang & Liu, 2001). PCR temperature profiles were the following for all three markers: Denaturation at 95 °C for 2 min., then 30 s. at 94 °C, 20 s. at 52 °C and 1 min. at 72 °C for 35 cycles. Final elongation was at 72 °C for 10 min. PCR product quality was assessed on a 1.5% agarose gel, then purified by NucleoSpin Gel and PCR Clean-up kit (Macherey-Nagel, Germany). The purified product was sequenced from both ends using a Big Dye Terminator v. 3.1 Cycle Sequencing kit (Applied Biosystem), based on the method of sanger sequencing with an ABI 3130 Genetic Analyzer machine.

### Bioinformatic and statistical analysis

Chromatograms were converted to FASTA file format with BioEdit Sequence Alignment Editor (Hall, 1999). The 699 bp long sequences of the D-loop region were aligned and analysed by the MegaX software (Kumar et al., 2018) with the ClustalW algorithm. Haplotypes diversity was calculated based on the polymorphic sites and haplotypes by using the DnaSP version 5 (Librado & Rozas, 2009) software. Pairwise FST values were calculated by using the MegaX software (Kumar et al., 2018). Different haplotypes were checked and compared with outgroup sequences from the National Centre for Biotechnology Information (NCBI GenBank) standard database (https://www.ncbi.nlm.nih.gov/) by nucleotide BLAST (Basic Local Alignment Search Tools). CoI results were compared to the sequences of the BOLD (Barcode of Life Data System) system (http://v3.boldsystems.org/). The network of the D-loop haplotypes was built using a median joining algorithm by using PopART software (Rohl, Bandelt & Forster, 1999; Clement et al., 2002). We used the closest related species (*C. auratus*, *C. a. buergeri*, *Carassius carassius*, *C. cuvieri*, *Cyprinus carpio carpio*) as reference sequences along with other *C. gibelio* haplotypes found in the literature. In case of Cytb based on the genetic distance and the phylogenetic tree was reconstructed by MegaX software with Neighbour Joining fitting with Kimura-2 parameter and 1,000 bootstrap replication. The references were the haplotypes described in *C. gibelio* neotypes by Kalous et al. (2012), as well as European sequences described by Takada et al. in the *Carassius auratus*-complex, where a European clade was identified based on the Cytb sequence (Takada et al., 2010).

## Results

### D-loop

**Figure 2:**
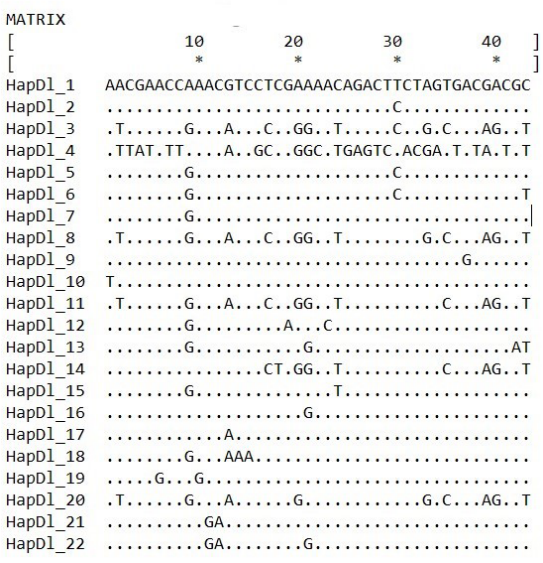
Hungarian *Carasssius gibelio* D-loop haplotypes and the polymorphic sites that define them.

**Figure 3:**
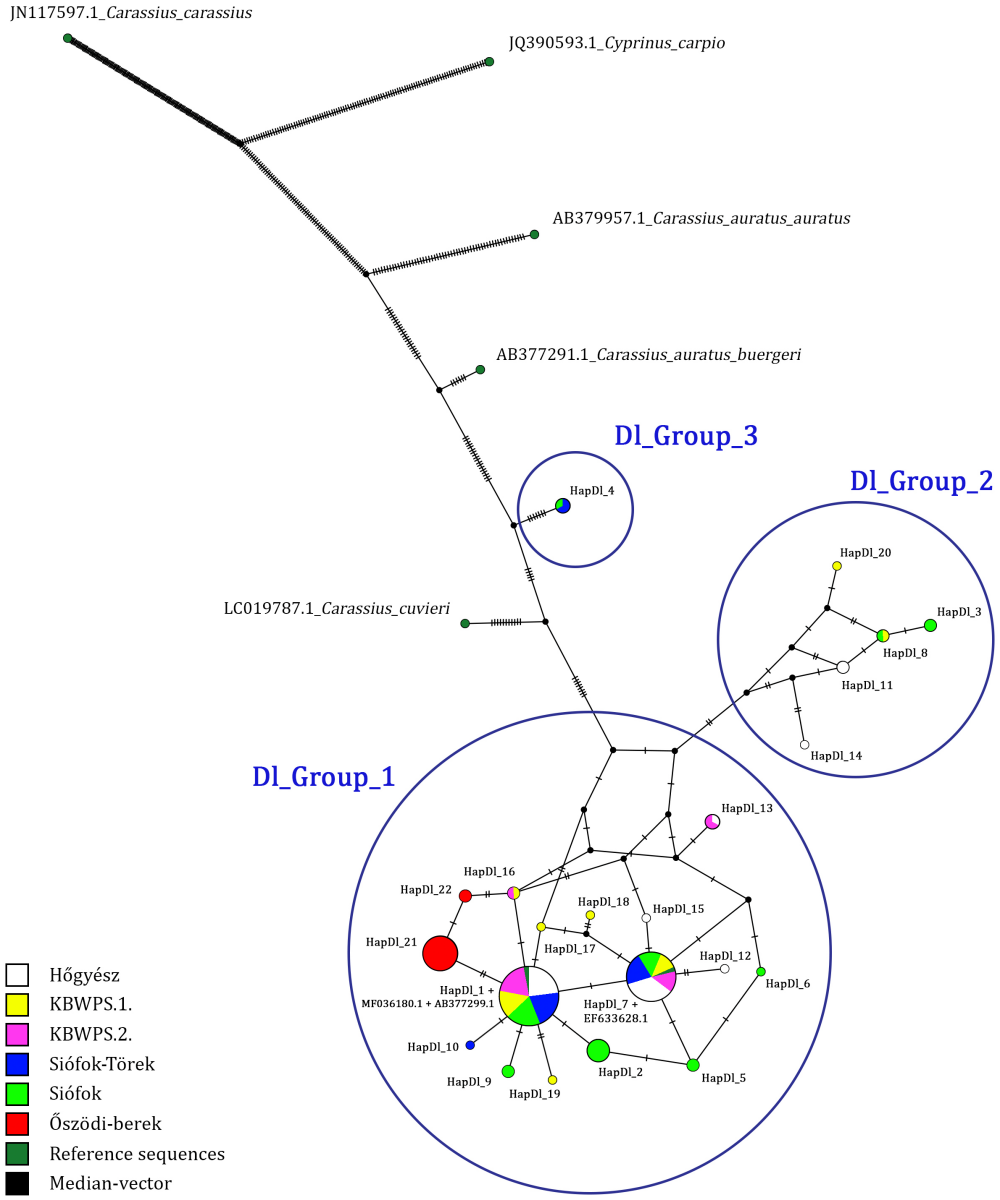
D-loop haplotype network obtained with PopART software, showing relationship among Hungarian Gibel carp populations. Black plots are the median vector inserted by PopART software and HapDl_n are the Hungarian haplotype groups. Blue circles are (Dl_Group 1, Dl_Group 2, Dl_Group 3) representing the haplotypes closest to eachother.

Based on the comparisons of 699 bp long sequences of the D-loop region, 22 haplotypes were identified in 132 individuals (Table 1). Three of these (HapDl_1, HapDl_7, HapDl_21) were identified in more than 15 individuals while the others were less frequent. These haplotypes were present in maximum 6 individuals. Within the haplotypes, the number of polymorphic sites was 43 (Fig. 2). For each population, the highest haplotype diversity was in Siófok 0,83 (HD) ± 0,04 (SD). The second was the KWBPS I with 0,80 (HD) ± 0,08 (SD). The lowest value was 0,19 (HD) ± 0,11 (SD) for the population of Őszödi-berek. In the population of Hőgyész HD was 0,72 ± 0,05 (SD), in the population of Siófok-Törek 0,66 ± 0,07 and in the population of KWBPS II. HD was 0,63 ± 0,09 (SD). Thirty-five sites were parsimony informative, based on the DnaSP analyses. Samples from all locations represented a mixture of different haplotypes except those for the sequences from Őszödi-berek, which separated into two individual groups (HapDl_21, HapDl_22) and the HapDl_2 group with seven individuals from Lake Balaton. Twelve other haplotypes were found (HapDl_3, HapDl_5, HapDl_6, HapDl_10, HapDl_11, HapDl_12, HapDl_14, HapDl_15, HapDl_17, HapDl_18, HapDl_19, HapDl_20), of which each contained only one or two samples from one location. The identified haplotypes were divided into three major groups separated on the network figure (Fig. 3). Most of the haplotypes, included the two largest groups (HapDl_1, HapDl_7), clustered together in the first group (Dl_Group 1.). The second group (Dl_Group 2.) contained five haplotypes (HapDl_3, HapDl_8, HapDl_11, HapDl_14, HapDl_20) and the third group contained only one haplotype (HapDl_4) closer to the *Carassius auratus buergeri* (AB377291.1) sequence, than the other *Carassius gibelio* haplotypes did. The rest of the outgroup sequences (AC.: LC019787.1, JN117597.1, JX122531.1, JQ390593.1) were separated according to the genetic distance.

**Table 1:** Gibel carp populations and the number of haplotypes identified in them.

	**N (Sio)**	**N (Hog)**	**N (KWBPS II.)**	**N (Ob)**	**N (KWBPS I.)**	**N (To)**	**N (all)**
HapDl_1	9	11	10	0	7	9	*46*
HapDl_2	7	0	0	0	0	0	*7*
HapDl_3	2	0	0	0	0	0	*2*
HapDl_4	1	0	0	0	0	2	*3*
HapDl_5	2	0	0	0	0	0	*2*
HapDl_6	1	0	0	0	0	0	*1*
HapDl_7	5	12	5	0	4	7	*33*
HapDl_8	1	0	0	0	1	0	*2*
HapDl_9	1	1	0	0	0	0	*2*
HapDl_10	0	0	0	0	0	1	*1*
HapDl_11	0	2	0	0	0	0	*2*
HapDl_12	0	1	0	0	0	0	*1*
HapDl_13	0	1	2	0	0	0	*3*
HapDl_14	0	1	0	0	0	0	*1*
HapDl_15	0	1	0	0	0	0	*1*
HapDl_16	0	0	1	0	1	0	*2*
HapDl_17	0	0	0	0	1	0	*1*
HapDl_18	0	0	0	0	1	0	*1*
HapDl_19	0	0	0	0	1	0	*1*
HapDl_20	0	0	0	0	1	0	*1*
HapDl_21	0	0	0	17	0	0	*17*
HapDl_22	0	0	0	2	0	0	*2*

**Notes.**

Sio Siófok Hog Hőgyész KBWPS II Kis-Balaton Water Protecting System II Stage, Ob Őszödi-berek KBWPS I Kis-Balaton Water Protecting System I Stage, To Törek and N (HapDl) the number of D-loop haplotypes

Six groups (HapDl_3, HapDl_4, HapDl_8, HapDl_9, HapDl_11, HapDl_15) with the total of 12 individuals were identified differently as gibel carp (Table 1). Two of them (HapDl_11, HapDl_15) contained only samples from Hőgyész and were identified as *C. auratus*. Samples from haplotype 8, 9, and 3 were identified as *C. auratus* too, along with samples from Lake Balaton and KBWPS Stage I. Haplotype 4 has shown the highest similarity with *C. a. buergeri* instead of gibel carps. Samples in this group were from Lake Balaton and the ponds of Siófok-Töreki. KBWPS Stage II. was the sole population with only *C. gibelio* haplotypes (EF633617.1, MF083605.1, MF036180.1, MF036179.1). Lake Balaton samples have the highest genetic diversity of nine haplotypes. This was followed by the samples from KBWPS Stage I. with eight haplotypes (Table 1). Őszödi-berek had only two haplotypes (HapDl_ 21, HapDl_22). The two D-loop sequence variants, detected in the case of 19 individuals characterized only this population.

Pairwise FST value (Table 2) among the populations has shown a moderate difference between each pair but most of the values were lower than 0.1, with the exception of one population (Őszödi-berek) which resulted in higher values. The lowest difference (−0.015) was identified between the populations of Hőgyész and KBWPS Stage I.

### Cytochrome *b*

**Figure 4:**
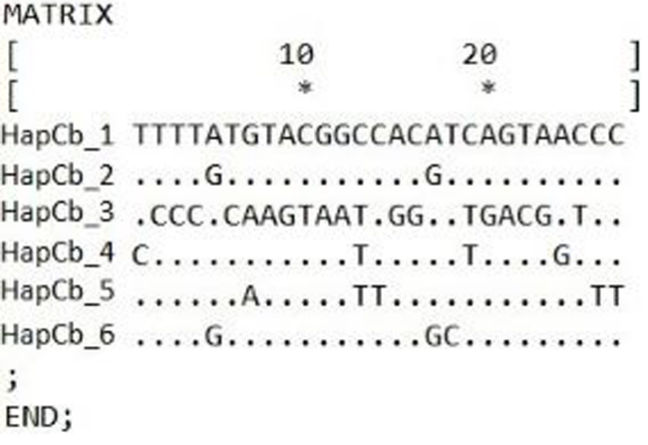
Hungarian Gibel carp Cytochrome *b* haplotypes and the defining polymorphic sites, made by DnaSp.

**Figure 5:**
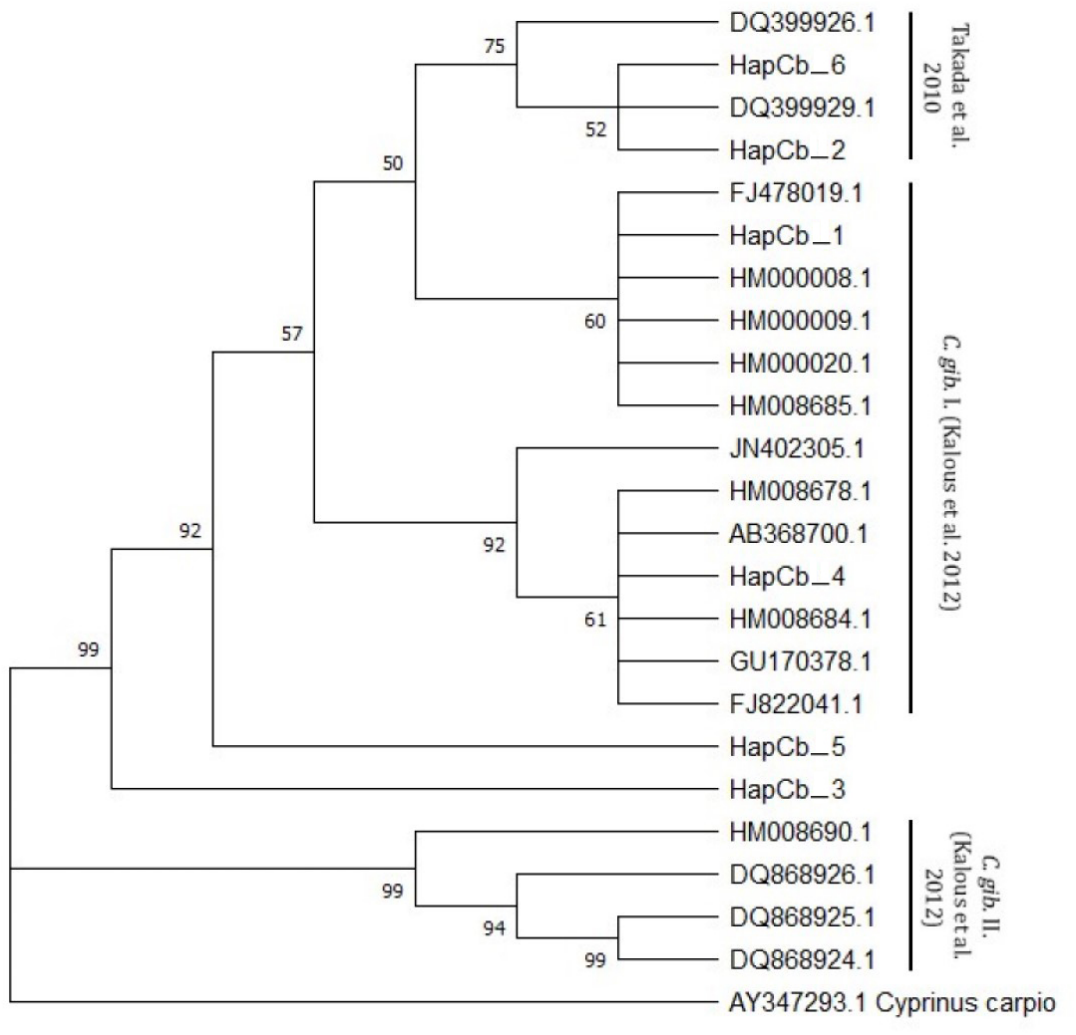
Cytb haplotypes compared to two gibel carp subgroups (C. gib. I and C. gib. II) defined by Kalous et al. and the sequences of Czech origins marked and designated as European clade by Takada et al. Phylogenetic tree was prepared with Neighbour-Joining fitting, taking into account the Kimura-2-parameter model, using a bootstrap value of 1000 with MEGA-X software, rooted a common carp Cytb sequence (AY347293. 1).

Cytb sequences were analysed to confirm the results of 22 D-loop haplotypes. Only six Cytb haplotypes were identified. The complete length of the sequence region was 1057 bp. Within the haplotypes the numbers of polymorphic sites were 27 (Fig. 4). NCBI database was used to check the evolutionary origin of the sequences. All six haplotypes were detected as *Carassius gibelio* (Table 3). None of the first 100 listed results are recognized as *C. auratus*, *C. a. buergeri* or other CAC species. Based on the previous work of Kalous et al. (2012) Cytb haplotypes were compared to the neotype sequences they described (AC: HM000009, HM0000020, GU170378, FJ822041, FJ478019, Ab368700, HM000008, HM008678, JN402305, HM008684, HM008685.1, DQ868924, DQ868925, DQ868926, HM008690) and the sequences described by Takada et al. (2010) (DQ399926.1., DQ399929.1) and were presented together on the phylogenetic tree (Fig. 5). Two of the six Hungarian haplotypes (HapCb_1, HapCb_4) were integrated into the first neotype group with other European sequences and two haplotypes to neotype II. (HapCb_3, HapCb_5) which contain only reference sequences from Mongolia. The third group on the top of the three contained the sequences described by Takada et al. (2010), and the Hungarian HapCb_6 and HapCb_2.

**Table 2:** Pairwise FST values between gibel carp populations created by MEGA X software.

	**Siófok-Töreki**	**Törek**	**Hőgyész**	**KWBPS II**	**KWBPS I**
**Siófok_Töreki**					
**Törek**	0.010				
**Hőgyész**	0.034	0.018			
**KWBPS II**	0.073	0.028	0.020		
**KWBPS I**	0.012	0.002	−0.015	0.017	
**Őszödi-berek**	0.485	0.423	0.616	0.751	0.556

**Table 3:** Gibel carp Cytb haplotype identification results by BLAST system.

	**BLAST**	**Query**	**Identity**	**Ac. number**
HapCb_1	*Carassius gibelio*	100%	100%	KX601122.1
HapCb_2	*Carassius gibelio*	100%	100%	HM000019.1
HapCb_3	*Carassius gibelio*	100%	100%	KX601124.1
HapCb_4	*Carassius gibelio*	100%	99%	HQ689899.1
HapCb_5	*Carassius gibelio*	100%	100%	MG281946.1
HapCb_6	*Carassius gibelio*	100%	99%	LC337602.1

### Cytochrome *c* oxidase 1

**Figure 6:**
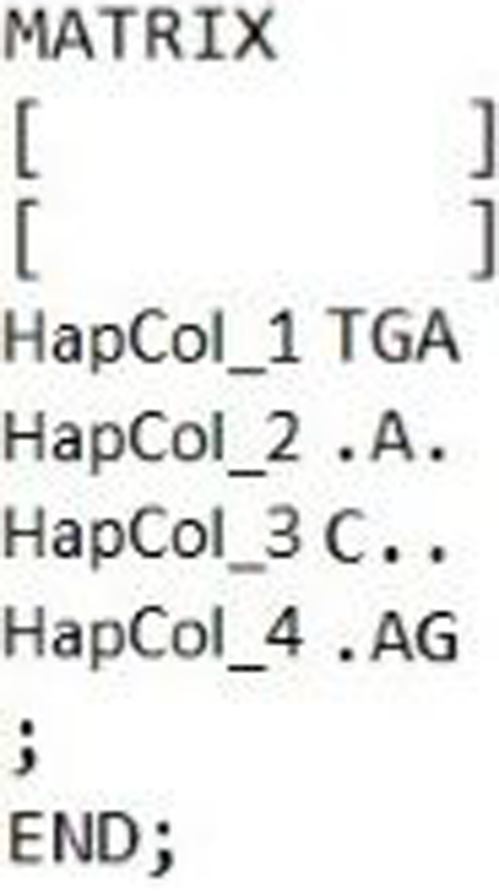
Hungarian gibel carp’s Cytochrome *c.* oxidase I haplotypes and the polymorphic sites defined them, made by DnaSp ver. 6.

CoI gene sequences of the 22 D-loop haplotypes were also determined. The length of the analysed fragment was 562 bp long. Four haplotypes (Fig. 6) were identified. Within the haplotypes the number of polymorphic sites was three. Sequences were analysed by NCBI BLAST and BOLD system. According to the BLAST standard nucleotide database two groups (HapCoI_2 and HapCoI_4) out of four showed the highest similarity with *Tatia intermedia*, one with *Cyprinus carpio haematopterus* (HapCoI_1) and one with *C. auratus* (HapCoI_3) (Table 4).

**Figure 7:**
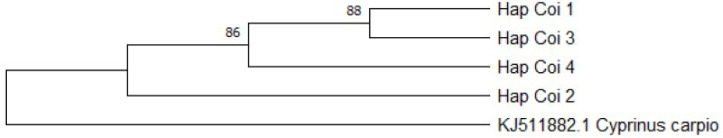
Gibel carp phylogenetic tree based on Cytochrome oxidase I haplotypes. Phylogenetic tree was prepared with Neighbour-Joining fitting, taking into account the Kimura-2-parameter model, using a bootstrap value of 1000 with MEGA-X software, rooted a common carp COI sequence (JX9832831).

The group containing most of the samples (HapCoI_2) had 100% of identity coverage of the *Tatia intermedia* query sequence. Furthermore, based on the BOLD system HapCoI_2 and HapCoI_4 were identified as *C. gibelio*. HapCoI_1, as well as HapCoI_3 have shown the highest similarity with *C. a. auratus*. In the case of HapCoI_3, not even the first 20 hits contained *C. gibelio* sequences. The phylogenetic tree formed three clearly visible branches (Fig. 7). The first branch included the HapCoI_1 and HapCoI_3. The second branch contained the HapCoI_2 and the HapCoI_4 was in the third group.

## Discussion

Sequence diversity of three mitochondrial loci (D-loop, Cytb, and CoI) was analysed in six natural *Carassius gibelio* populations in Hungary. Among the 132 aligned D-loop sequences 22 haplotypes were identified. The majority of the individuals belonged to two haplotypes (HapDl_1 and HapDl_7), characterizing almost every sampling location. These haplogroups contained samples also from the geographically distant fish ponds of Hőgyész, which is attributed to be ancestral. These haplotypes are in a centroid position among others in the network system (Fig. 3). The rest of the haplotypes were found only in a small number of individuals.

**Table 4:** Gibel carp CoI haplotype identification results by BLAST and BOLD system.

**CoI**	**BLAST results**	**Accession number**	**BOLD results**
HapCoI_1 (*n* = 1)	*Cyprinus carpio haematopterus*	MK726305.1	*Carassius auratus*
HapCoI_2 (*n* = 17)	*Tatia intermedia*	MK078120.1	*Carassius gibelio*
HapCoI_3 (*n* = 3)	*Carassius auratus auratus*	MT559523.1	*Carassius auratus*
HapCoI_4 (*n* = 1)	*Tatia intermedia*	MK078120.1	*Carassius gibelio*

The largest number of haplotypes of all three loci were found in Lake Balaton, which corresponds to the size of the lake (the largest shallow lake in Central Europe Istvanovics et al., 2007). Haplotype diversity is potentially corresponded with the diverse and geographically isolated spawning habitats, situated in the inflows throughout the catchment. Young fish usually stay in these habitats until they reach 1–3 years of age (Specziár, 2010). Smaller populations contained less haplotypes, ranging from two (Őszödi-berek) to eight (Hőgyész).

Based on the fixation index, the differences among populations are limited. This indicates the presence of gene flow because of the migration and efficient reproduction among the populations. The only exception is the population of Őszödi-berek, which is a completely closed water system, no fish can reach or escape from it. There, we identified only two haplotypes and they were not present in other populations. This wetland was disconnected from other water bodies during the water regulation period of the early 19th century (Zlinszky & Timar, 2013). Gibel carp was most possibly introduced to this site unintentionally by coarse angels, which has been documented in other systems in the earlier years (Docherty et al., 2017). The wetland suffered from multiple, at least partial droughts in the last 20 years, which most possibly resulted in a population level bottleneck (Ferincz et al., 2016a; Lennox et al., 2019). Similar observations were made in the cases of *Gadopsis marmoratus* in Australia (Coleman et al., 2018) and *Eupallasella percnurus* in Poland (Kaczmarczyk & Wolnicki, 2016), which reinforced that isolated populations are more vulnerable to stochastic events.

Five *C. a. auratus* (HapDl_1, HapDl_3, HapDl_8, HapDl_14, HapDl_20) and one *C. a. buergeri* (HapDl_4) haplotypes were identified in 5 different populations with low frequency. The most distant haplotype (HapDl_4) was identified as *C. a. buergeri* (Temminck & Schlegel, 1846) but contained only 3 individuals. The genetic detachment of these individuals is clear. The presence of these haplotypes in the studied natural “gibel carp” populations clearly indicates interspecific hybridization among CAC species in this region. The two ornamentally bred species (*C. auratus* and *C. a. buergeri*) are not native in the European waters, but originated from different types of goldfish kept commonly by aquarists. None of the previous European studies (Kalous et al., 2012; Rylkova & Kalous, 2013; Kalous et al., 2007; Tsipas et al., 2009) revealed the presence of *C. a. buergeri* from natural waters outside of Asia (Ueda & Ojima, 1978; Ojima & Yamano, 1980; Kobayasi, Ochi & Takeuchi, 1973). Our research did not find any sequence that could originate from other nonindigenous species in CAC previously described from European waters (*e.g.*, *Carassius langsdorfii)* (Rylkova & Kalous, 2013; Kalous et al., 2007; Tsipas et al., 2009) or hybrids with the native *Carassius carassius* (Zlinszky & Timar, 2013; Rylkova et al., 2013). Nonetheless the hybridization between the members of the CAC cannot be ruled out based solely on the mitochondrial regions. Because of their maternal inheritance, only the female *Carassius gibelio* sequences are detectable and the paternal lines remains hidden.

To get a more comprehensive information, the sequences of Cytb and CoI loci of 22 haplotypes were examined. However, neither Cytb nor CoI has shown as many haplotypes as the D-loop did. Rylkova & Kalous (2013) also reported that Cytb showed low genetic diversity. The Cytb haplotypes supported the phenotypic identification but does not confirm the presence of *C. auratus* and the group, determined as *C. a. buergeri*. Comparing to the D-loop BLAST results none of the first 100 scored Cytb hits contained sequences from the species *C. a. buergeri*. The CoI was the less informative marker of the three tested types. It has identified only four haplotypes. However, based on the BLAST analysis of the standard nucleotide full database of GeneBank, all four showed unreliable results. The highly scored overlapping with the driftwood catfish (*Tatia intermedia*) and the amur wild carp (*Cyprinus carpio haematopterus*) in the database draws attention to the weakness of using online databases, which could contain incorrectly uploaded data. For example Elgin, Tunna & Jackson (2014) and Buhay (2009) both had similar experience with the CoI. The benefits of the BOLD (barcode based) system were emphasized, but it did not help to clarify our results. Based on our results, we agree with Tsipas et al. (2009) that in the future, in order to determine the status of gibel carp in taxonomic studies, it will be necessary to include nuclear markers in addition to mitochondrial markers. Nuclear genetic markers are able to widen genetic identification and may be suitable for finding foreign sequence pieces that can be used to explore the hybrid origin, which may be hidden due to maternal inheritance in the analyses of the mitochondrial genome. However, more markers (from 4 to 70) should have to be used for the proper identification of hybrids in the later or backcrossed generations (Boecklen & Howard, 1997).

The Cytb and CoI have the lowest mutation rate among the mitochondrial protein coding genes in fish. The CoI is used in BOLD system for species identification but our results are revealed that Cytb can be used more efficiently for identification the CAC complex because of more reliable databases. This marker showed the highest agreement with the phenotypes. While the D-loop showed the highest genetic variability. It is in agreement with other studies and explained by the highest mutation rate of the only larger non-coding mitochondrial region (Brown Wesley, Matthew Jr & Wilson, 1979; Parker et al., 1998). It is much more informative, than the nuclear markers when used for the analysis of closely related species, subspecies categories or populations (Brown Wesley, Matthew Jr & Wilson, 1979; Lagouge & Larsson, 2013; Ballard & Whitlock, 2004). Our results also show that this region can be efficiently used for intrapopulation analyses for identifying hybrids in CAC.

## Conclusions

This study is the first genetic diversity assessment for Hungarian gibel carp populations, in which we reported the recent homogenous genetic background of the studied populations. However, the potential hybrid origins of gibel carps were identified in the studied waters. The origin of the introgressed *C. auratus* sequences is doubtful but denotes the unreliability of morphological based identification of taxa within the genus *Carassius* and the hidden presence of goldfish in natural waters of Hungary.

## Supplemental Information

10.7717/peerj.12441/supp-1 Supplemental Information 1 Aligned Cytochrome b. haplotypes in FASTA format

10.7717/peerj.12441/supp-2 Supplemental Information 2 Aligned Cytochrome oxidase haplotypes in FASTA format

10.7717/peerj.12441/supp-3 Supplemental Information 3 Aligned D-loop haplotypes in FASTA format

## Additional Information and Declarations

### Field Study Permissions

The following information was supplied relating to field study approvals (i.e., approving body and any reference numbers): Tissue sampling was authorized by the Minister of Agriculture (Permit no.: HHgF/122-1/2018).

### Data Availability

The following information was supplied regarding data availability: All alignments are available in the Supplemental Files. The haplotype sequences are available at GenBank: MW379849 to MW379880.

## References

[ref-1] Ballard JWO, Whitlock MC (2004). The incomplete natural history of mitochondria. Molecular Ecology.

[ref-2] Balon EK, Blanc M, Banarescu P, Gaudet J-L, Hureau J-C (1974). European Inland water fish. A multilingual catalogue. Copeia.

[ref-3] Berg LS (1932). Übersicht der Verbreitung der Süß wasserfische Europas. Zoogeoraphica.

[ref-4] Boecklen WJ, Howard DJ (1997). Genetic analysis of hybrid zones: numbers of markers and power of resolution. Ecology.

[ref-5] Brown Wesley M, Matthew Jr George, Wilson AC (1979). Rapid evolution of animal mitochondrial DNA. Proceedings of the National Academy of Sciences of the United States of America.

[ref-6] Bănărescu P (1991). Distribution and dispersal of freshwater animals in North America and Eurasia. AULA-Verlag Wiesbaden Germany.

[ref-7] Buhay JE (2009). Coi-like sequences are becoming problematic in molecular systematic and dna barcodeng studies. Journal of Crustacean Biology.

[ref-8] Clement M, Snell Q, Walke P, Posada D, Crandall K (2002). TCS: estimating gene genealogies. Proc. - Int. Parallel Distrib. Process. Symp. IPDPS.

[ref-9] Coleman RA, Gauffre B, Pavlova A, Beheregaray LB, Kearns J, Lyon J, Sasaki M, Leblois R, Sgro C, Sunnucks P (2018). Artificial barriers prevent genetic recovery of small isolated populations of a low-mobility freshwater fish. Heredity (Edinb).

[ref-10] De Giosa M, Czerniejewski P, Rybczyk A (2014). Seasonal changes in condition factor and weight-length relationship of invasive *Carassius gibelio* (Bloch, 1782) from Leszczynskie Lakeland, Poland. Journal of Advanced Zoology.

[ref-11] Docherty C, Rupert J, Tyana R, Andreas H, Poesch MS (2017). Assessing the spread and potential impact of Prussian Carp *Carassius gibelio* (Bloch, 1782) to freshwater fishes in western North America Cassandra. BioInvasions Records.

[ref-12] Elgin EL, Tunna HR, Jackson LJ (2014). First confirmed records of Prussian carp. Carassius gibelio (Bloch, 1782) in open waters of North America. BioInvasions Records.

[ref-13] Ferincz Á, Horváth Z, Staszny Á, Ács A, Kováts N, Vad CF, Csaba J, Sütő S, Paulovits G (2016a). Desiccation frequency drives local invasions of non-native gibel carp (*Carassius gibelio*) in the catchment of a large, shallow lake (Lake Balaton, Hungary). Fisheries Research.

[ref-14] Ferincz Á, Staszny Á, Weiperth A, Takács P, Urbányi B, Vilizzi L, Paulovits G, Copp GH (2016b). Risk assessment of non-native fishes in the catchment of the largest Central-European shallow lake (Lake Balaton, Hungary). Hydrobiologia.

[ref-15] Guti G (1993). A magyar halfauna természetvédelmi minősítésére javasolt értékrendszer. HaláSzat.

[ref-16] Halačka K, Lusková V, Lusk S (2003). Carassius gibelio in fish communities of the Czech Republic. Ecohydrology & Hydrobiology.

[ref-17] Hall TA (1999). BioEdit: a user-friendly biological sequence alignment editor and analysis program for Windows 95/98 NT. Nucleic Acids Symposium Series.

[ref-18] Harper LR, Griffiths NP, Lawson Handley L, Sayer CD, Read DS, Harper KJ, Blackman RC, Li J, Hänfling B (2019). Development and application of environmental DNA surveillance for the threatened crucian carp (*Carassius carassius*). Freshwater Biology.

[ref-19] Herman O (1887). A magyar halaszat könyve.

[ref-20] Holcík J (1980). Carassius auratus (Pisces) in the Danube river. Acta Scientiarum Naturalium - Academiae Scientiarum Bohemoslovacae.

[ref-21] Istvánovics V, Clement A, Somlyódy L, Specziár A, G.-Tóth L, Padisák J (2007). Updating water quality targets for shallow Lake Balaton (Hungary), recovering from eutrophication. Hydrobiologia.

[ref-22] Ivanova NV, Zemlak TS, Hanner RH, Hebert PDN (2007). Universal primer cocktails for fish DNA barcoding. Molecular Ecology Resources.

[ref-23] Kaczmarczyk D, Wolnicki J (2016). Genetic diversity of the critically endangered lake minnow *Eupallasella percnurus* in Poland and its implications for conservation. PLOS ONE.

[ref-24] Kalous L, Bohlen J, Rylková K, Petrtý M (2012). Hidden diversity within the Prussian carp and designation of a neotype for *Carassius gibelio* (Teleostei: Cyprinidae). Ichthyological Exploration of Freshwaters.

[ref-25] Kalous L, Knytl M (2011). Karyotype diversity of the offspring resulting from reproduction experiment between diploid male and triploid female of silver prussian carp, carassius gibelio (Cyprinidae, Actinopterygii). Folia Zoologica.

[ref-26] Kalous L, Šlechtová V, Bohlen J, Petrtýl M, Švátora M (2007). First european record of *Carassius langsdorfii* from the Elbe basin. Journal of Fish Biology.

[ref-27] Kobayasi H, Ochi H, Takeuchi N (1973). Chromosome studies in the genus *Carassius*: comparison of *C. auratus grandoculis*, *C. auratus buergeri*, and *C. auratus langsdorfii*. The Japanese Journal of Ichthyology.

[ref-28] Kumar S, Stecher G, Li M, Knyaz C, Tamura K (2018). MEGA X: molecular evolutionary genetics analysis across computing platforms. Molecular Biology and Evolution.

[ref-29] Lagouge M, Larsson NG (2013). The role of mitochondrial DNA mutations and free radicals in disease and ageing. Journal of Internal Medicine.

[ref-30] Lelek A (1987). Threatened fishes of Europe. Freshwater Fishes of Europe 9.

[ref-31] Lennox RJ, Crook DA, Moyle PB, Struthers DP, Cooke SJ (2019). Toward a better understanding of freshwater fish responses to an increasingly drought-stricken world. Reviews in Fish Biology and Fisheries.

[ref-32] Librado P, Rozas J (2009). DnaSP v5: a software for comprehensive analysis of DNA polymorphism data. Bioinformatics.

[ref-33] Nelson SJ, Grande T, Wilson MVH (1994). Fishes of the world. Third edition.

[ref-34] Ojima Y, Yamano T (1980). The assignment of the nucleolar organizer in the chromosomes of the funa (Carassius, Cyprinidae, Pisces). Proceedings of the Japan Academy, Series B.

[ref-35] Parker PG, Snow AA, Schug MD, Booton GC, Fuerst PA (1998). What molecules can tell us about populations: choosing and using a molecular marker. Ecology.

[ref-36] Perdikaris C, Ergolavou A, Gouva E, Nathanailides C, Chantzaropoulos A, Paschos I (2012). Carassius gibelio in Greece: the dominant naturalised invader of freshwaters. Reviews in Fish Biology and Fisheries.

[ref-37] Pintér K (2002). Magyarország halai.

[ref-38] Piria M, Povž M, Vilizzi L, Zanella D, Simonović P, Copp GH (2016). Risk screening of non-native freshwater fishes in Croatia and Slovenia using the Fish Invasiveness Screening Kit. Fisheries Management & Ecology.

[ref-39] Przybył A, Przybylski M, Spóz A, Juchno D, Szabelska A, Kowalewska K, Boroń A (2020). Sex, size and ploidy ratios of *Carassius gibelio* from poland. Aquatic Invasions.

[ref-40] Puntila R, Vilizzi L, Lehtiniemi M, Copp GH (2013). First application of FISK, the freshwater fish invasiveness screening kit, in northern Europe: example of southern Finland. Risk Analysis.

[ref-41] Röhl A, Bandelt HJ, Forster P (1999). Median-joining networks for inferring intraspecific phylogenies. Molecular Biology and Evolution.

[ref-42] Rylková K, Kalous L (2013). Genetic diversity in the genus *Carassius* (Teleostei: Cyprinidae) in the Czech Republic. Acta Societatis Zoologicae Bohemicae.

[ref-43] Rylková K, Kalous L, Bohlen J, Lamatsch DK, Petrtýl M (2013). Phylogeny and biogeographic history of the cyprinid fish genus *Carassius* (Teleostei: Cyprinidae) with focus on natural and anthropogenic arrivals in Europe. Aquaculture.

[ref-44] Rylková K, Petrtýl M, Bui AT, Kalous L (2018). Just a Vietnamese goldfish or another *Carassius*? Validity of *Carassius argenteaphthalmus* Nguyen & Ngo, 2001 (Teleostei: Cyprinidae). Journal of Zoological Systematics and Evolutionary Research.

[ref-45] Specziár A (2010). A Balaton halfaunája: a halállomány összetétele, az egyes halfajok életkörülményei és a halállomány korszerű hasznosításának feltételrendszere. Acta Biologica Debrecina. Supplementum Oecologica Hungarica.

[ref-46] Szalay M (1954). Új halfaj Magyarországon. Halászat.

[ref-47] Takács P, Czeglédi I, Ferincz Á, Sály P, Specziár A, Vitál Z, Weiperth A, Erős T (2017). Non-native fish species in Hungarian waters: historical overview, potential sources and recent trends in their distribution. Hydrobiologia.

[ref-48] Takada M, Iguchi K, Nishida M, Miya M, Tachihara K, Kon T (2010). Biogeography and evolution of the *Carassius auratus*-complex in East Asia. BMC Evolutionary Biology.

[ref-49] Temminck CJ, Schlegel H (1846). Pisces. Fauna Japonica, sive descriptio animalium quae in itinere per Japoniam suscepto annis 1823-30 collegit, notis observationibus et adumbrationibus illustravit P. F. de Siebold,. Parts 10–14.

[ref-50] Toth B, Váradi L, Várkonyi E, Hidas A (2000). Silver crucian carp (carassius auratus gibelio blocH, X X X ) in the danube river basin. Tiscia monograph series.

[ref-51] Tóth B, Várkonyi E, Hidas A, Meleg E, Váradi L (2005). Genetic analysis of offspring from intra- and interspecific crosses of *Carassius auratus gibelio* by chromosome and RAPD analysis. Journal of Fish Biology.

[ref-52] Tsipas G, Tsiamis G, Vidalis K, Bourtzis K (2009). Genetic differentiation among Greek lake populations of *Carassius gibelio* and *Cyprinus carpio carpio*. Genetica.

[ref-53] Ueda T, Ojima Y (1978). Differential chromosomal characteristics in the funa subspecies (Carassius). Proceedings of the Japan Academy, Series B.

[ref-54] Vasile EL, Gibelio IC, Species N (2019). EL vasile* is *Carassius Gibelio* (Pisces, Cyprinidae) a native or non-native species in romania?. Scientific Annals of the Danube Delta Institute.

[ref-55] Verreycken H, Anseeuw D, Van Thuyne G, Quataert P, Belpaire C (2007). The non-indigenous freshwater fishes of Flanders (Belgium): review, status and trends over the last decade. Journal of Fish Biology.

[ref-56] Wang C, Li S, Nagy ZT, Lehoczky I, Huang L, Zhao Y, Song X, Jeney Z (2010). Molecular genetic structure and relationship of Chinese and Hungarian common carp (*Cyprinus carpio L.*) strains based on mitochondrial sequence. Aquaculture Research.

[ref-57] Wouters J, Janson S, Lusková V, Olsén KH (2012). Molecular identification of hybrids of the invasive gibel carp *Carassius auratus gibelio* and crucian carp *Carassius carassius* in Swedish waters. Journal of Fish Biology.

[ref-58] Xiao W, Zhang Y, Liu H (2001). Molecular systematics of Xenocyprinae (Teleostei: Cyprinidae): taxonomy, biogeography, and coevolution of a special group restricted in East Asia. Molecular Phylogenetics and Evolution.

[ref-59] Zhou L, Gui J (2017). Natural and artificial polyploids in aquaculture. Aquaculture and Fisheries.

[ref-60] Zhou L, Wang Y, Gui JF (2000). Genetic evidence for gonochoristic reproduction in gynogenetic silver crucian carp (*Carassius auratus gibelio* Bloch) as revealed by RAPD assays. Journal of Molecular Evolution.

[ref-61] Zlinszky A, Timár G (2013). Historic maps as a data source for socio-hydrology: a case study of the Lake Balaton wetland system, Hungary. Hydrology and Earth System Sciences.

